# How to Improve Others’ Emotions: Reappraise and be Responsive

**DOI:** 10.1007/s42761-023-00183-4

**Published:** 2023-04-15

**Authors:** Olivia Jurkiewicz, C. Blair McGarrigle, Christopher Oveis

**Affiliations:** 1 University of California, San Diego, USA; 2 University of California, Los Angeles, USA

**Keywords:** Interpersonal emotion regulation, Reappraisal, Responsiveness, Social support, Social regulation

## Abstract

People often try to improve others’ emotions. However, it is unclear which interpersonal emotion regulation strategies are most effective and why. In 121 candid dyadic conversations between undergraduate students via video conferencing, target participants recounted a stressful event to regulator participants. Three strategies used by regulators during these conversations to change targets’ emotions were obtained from the regulator after the conversation: extrinsic reappraisal, extrinsic suppression, and extrinsic acceptance. Perceived regulator responsiveness was obtained from targets to examine the social consequences of extrinsic emotion regulation and its mediating role in successful extrinsic emotion regulation. We found that regulators’ extrinsic reappraisal use was associated with improved target emotions measured across two distinct classes of outcomes: targets’ emotions during the conversation and targets’ perception that the regulator improved their emotions. Regulators’ extrinsic suppression and acceptance, in contrast, were not related with improved target emotions or perceptions of improvement. Instead, all extrinsic regulatory strategies were associated with improved targets’ emotions when mediated by targets’ perceptions of regulator responsiveness. Finally, observer-ratings of regulators’ extrinsic reappraisal and suppression use were found to be consistent with regulators’ self-ratings and follow the same pattern of results on the outcome measures. These findings provide insight into why the social regulation of emotions can succeed or fail and hold implications for interventions aimed at guiding people toward more successfully improving others’ emotions.

**Supplementary Information:**

The online version contains supplementary material available at 10.1007/s42761-023-00183-4.

Overwhelmed with his new job, Tyler divulges his fears and doubts to his coworker Rebecca. Tyler fears failing the people who gave him this job opportunity, and he doubts that he can keep up with the accumulating workload. When Rebecca hears about his distress, she considers using three different strategies to help him. She could remind him that many people struggle at first in a new job, so it is okay to feel this way (i.e., extrinsic acceptance), she could scold him for being so negative at work because it looks bad for him (i.e., extrinsic suppression), or she could suggest that he reframe his situation as a learning experience in which it is okay to make mistakes (i.e., extrinsic reappraisal). Ultimately, she chooses the latter strategy, which proves effective in easing Tyler’s negative emotions.

This example highlights the importance of interpersonal emotion regulation, a process in which one person’s emotions are regulated by another person (Dixon-Gordon et al., 2015; Zaki & Williams, 2013). Most emotion regulation research prioritizes understanding how the individual uses strategies to change their own emotion experience (Gross, 2015) but overlooks the role of other people in this process (Reeck et al., 2016). Yet, it is well documented that other people have the capacity to change a target person’s emotions through their communication style (Overall et al., 2009), their emotion expression (Parkinson & Simons, 2009; Peters et al., 2014), their physical proximity (Beckes & Sbarra, 2022), and their empathic ability (Brown & Fredrickson, 2021). That is why individuals like Tyler often depend on others to improve how they feel (Williams et al., 2018).

The process of interpersonal emotion regulation may be initiated by the target (i.e., intrinsic) as in the case of the social sharing of emotions (Rimé, 2009) or may be initiated by regulator (i.e., extrinsic; Niven, 2017) as in the example of Rebecca and Tyler. Some conceptualizations specify that interpersonal emotion regulation must be deliberate (Niven et al., 2009), and other work does not. We take the position here that extrinsic emotion regulation may be either deliberate or non-deliberate on the part of the regulator. Thus far, research on extrinsic emotion regulation has demonstrated that individuals choose to use extrinsic reappraisal (Matthews et al., 2021) and extrinsic suppression (Pauw et al., 2019) on others to intentionally modify their emotions, and that this is a daily occurrence (Liu et al., 2021). In the present study, we examine whether three extrinsic strategies (i.e., reappraisal, suppression, and acceptance) might work and why by capturing natural behavior in candid conversations in which authentic emotions are experienced.

Similar to intrapersonal cognitive reappraisal which works through the reinterpretation of an emotion-eliciting situation (Gross, 1998, 2015), extrinsic reappraisal should be effective when a regulator suggests a suitable reframing of the target’s situation. An advantage of extrinsic reappraisal is that the regulator provides additional cognitive resources to the target, thereby reducing the effort needed by the target to formulate their own alternative interpretations (for a discussion on load sharing, see Coan & Sbarra, 2015). Because reappraisal is a difficult strategy for individuals to successfully execute on their own due to cognitive load (Ford & Troy, 2019), any external support may be useful. For example, listening to another’s reinterpretation of a negative image bolsters better intrapersonal emotion regulation (Sahi et al., 2021). On the other hand, there are reasons to assume that extrinsic reappraisal would not be effective, particularly if the target does not accept the cognitive reframing chosen by the regulator. For example, targets perceive written advice from regulators about using reappraisal as unhelpful for managing their anxiety compared to managing their sadness (Shu et al., 2020). One possible explanation is that targets can feel offended or demeaned by a regulator’s reinterpretation of their emotion experience, especially in the context of high arousal negative emotions such as anxiety and anger (Levenson et al., 2015).

When using extrinsic suppression, a regulator signals for a target person to hide their emotion expression through indirect means (e.g., ignoring or conveying discomfort about their emotional expressions) or direct means (e.g., telling them to hide or control their emotions). By suppressing a target’s expressions of negative emotions, a regulator may improve a target’s emotions by preventing detrimental social behaviors such as co-rumination (Boren, 2014) or venting (Nils & Rimé, 2012), or a regulator may improve a target’s emotions by shifting the target’s attention away from the distressing situation (Bebko et al., 2011). On the other hand, extrinsic suppression may result in the intensification of negative emotions due to unresolved negative appraisals (Yih et al., 2019) and the experience of new negative emotions due to the distressing social interaction (Lakey et al., 1994).

In the case of extrinsic acceptance, we conceptualize this strategy as one in which a regulator embraces the emotions of the target without attempts to control the target’s emotions which may or may not result in the target also accepting their own emotions. Extrinsic acceptance may result in greater emotion awareness and expression (Stanton & Low, 2012; Torre & Lieberman, 2018) or feelings of validation (Paivio & Laurent, 2001), but extrinsic acceptance may be unhelpful if the underlying negative appraisals are not effectively resolved or if the regulator does not also engage in acceptance behaviors (i.e., helping the target accept their own emotions).

We propose that extrinsic emotion regulation strategies function on both the emotions of the target through shifting their appraisals and the target’s social perceptions of the extrinsic strategies. First, by using an extrinsic strategy, a regulator provides a target with emotion regulation instructions or signals (e.g., to rethink, hide, or accept how they feel); these instructions/signals can influence the target’s cognitions and regulatory actions, potentially improving the target’s emotions. For example, a regulator may try to help a target downregulate their negative emotions due to a recent breakup by telling them that they “will regret” feeling like this later (i.e., extrinsic suppression, see Table 1), but if the extrinsic strategy does not effectively change the target’s underlining appraisal (e.g., fear of being alone), we propose that the strategy will not work. Therefore, we expect that extrinsic reappraisal will improve targets’ emotions by shifting appraisals, whereas extrinsic acceptance and suppression will not.

**Table 1:** Examples of regulator extrinsic strategy use from stress sharing conversations

Extrinsic reappraisal	R: *See, that brought you closer to your mom… there’s always good in whatever happens. This is what I believe.* R: *I think this is a positive experience no matter what you decide to do in the future.* R:* This time you are going to know what is expected and how the class is, so you will be better prepared.* R: *Each person’s abilities are different…maybe you have some other skills that they don’t have…you deal with other problems in your life better than them.* R: *The market now is just bad. Don’t feel too bad about yourself. It’s not like they don’t want to hire you. Some companies just go under, and they can’t hire anyone now.* R: [About social distancing] *It’s frustrating, but I feel like in the end it’s worth it just so they don’t get sick. So no one does.* R: *That’s why I think it’s important to not compare yourself with other people because you don’t know what their situation is.*
Extrinsic acceptance	R: *That’s stressful as hell. It really is. The job search is demoralizing, just entering resumes just over and over again into different websites that could’ve just been standardized. It’s tough.* R: *Oh my god. You’re in the 20 series. I don’t know the professors, but I just know the 20 series is super hard.* R: *It’s completely okay if you feel frustrated because it’s obviously a frustrating situation.* R: *I just wanted to say I’m really sorry you had to go through that. It’s a really tough situation.* R:* Yeah, I can imagine that for sure. How you prepare yourself… is definitely a stress I think many college students are dealing with.* R:* I think it totally sucks that you have to cut down on work hours, because the fact that you have a job means that you need money.* R: *I can’t imagine how hard it is for you. But I mean you’re getting through it. It’s been half a year, more than half a year now, so that’s really commendable.*
Extrinsic suppression	R: *Please focus on your studies because I think that’s the most important thing right now. You’ll be unhappy for a while but I think you will regret it after like six months because “I wasted my time on someone who broke my heart.”* R: *I feel like a lot of people are also going through what you’re going through* [said dismissively]. R: *Yeah uncertainty, that’s pretty much all of college students* [downplaying the target’s situation]. R: *I mean I’m sure you did okay.* R: *You’ll get over it.* T:* I’m a second year so I already put time and effort into this so I have to stick with it.* [Regulator changes subject to themself]* R: Yeah, for me…* [Target is stressed about financial situation] R: *Oh I see…I hope you get a job by then.* [Regulator does not add any more]. R: *I guess you’re experiencing a lot more stress than me… but I guess you’re in a much better position than me to know what to expect.*

Each example was selected from a different regulator who rated themselves in the upper 25th percentile of using the respective extrinsic strategy. These quotes are edited for grammar and clarity. *R*, regulator; *T*, target. View examples of these interactions at this link: https://osf.io/jmz69?view_only=fdac5950608a425aa273ba07ccd377ea

Secondly, we expect that these extrinsic strategies have social consequences which should impact whether they improve a target’s emotions. For example, when a regulator demonstrates their willingness to support the target’s emotional needs by taking the time and effort to help them reappraise, the target is likely to perceive the regulator as highly responsive (Reis & Shaver, 1988). Perceived responsiveness includes feeling that one is cared for, understood, and valued and is critical for building and maintaining healthy relationships (Gordon & Chen, 2016; Reis, 2017). In addition to these social perceptions, perceiving one’s social partner as responsive can improve one’s emotions (Maisel & Gable, 2009) by establishing a safe and positive social environment (Kane et al., 2012). Thus, we propose that perceived responsiveness should serve as one key social mechanism through which extrinsic action can improve a target’s emotions. In our theorizing, extrinsic reappraisal and acceptance, but not suppression, should result in enhanced perceived responsiveness because they necessarily involve demonstrating an understanding of and concern for the target.

In the present study, we examined whether extrinsic reappraisal, acceptance, and suppression predicted improved target emotions—and whether perceived responsiveness might mediate improvements in target emotions—by examining naturally occurring attempts at extrinsic emotion regulation during emotional conversations between newly acquainted target and regulator participants. We chose to measure these well-studied strategies because they may have different emotion outcomes interpersonally depending on their social consequences (Niven, in press). For instance, if extrinsic reappraisal is associated with worse perceived regulator responsiveness, we would also expect it to be associated with worse target affect. To determine target emotion improvement, we measured two types of outcomes from the targets: their emotions during the conversation and their perception of the regulators’ success at improving how they felt*.* We hypothesized that greater use of extrinsic reappraisal would predict target emotion improvement, and extrinsic acceptance and suppression would not. We also measured the target’s perception of the regulator’s responsiveness, hypothesizing that targets’ perceived regulator responsiveness would mediate the relationship between the extrinsic strategies and target emotion improvement.

## Method

### Participants

Two hundred seventy-two undergraduate students at the University of California, San Diego participated in this social interaction study in same-gender dyads. Participants received course credit. A power analysis, using G*Power, indicated that a sample size of 120 dyads (40 dyads per cell) would be sufficient to detect a medium effect size of *f* = 0.3 with 80% power. We thus aimed to collect data from at least 120 dyads and more if possible, terminating data collection at the conclusion of the academic quarter. Twenty-six participants were excluded due to internet connectivity issues or other technical issues during the study. Four participants were excluded due to knowing the other participant in the dyad. All other participants were paired with stranger participants. The final sample consisted of 242 participants (121 dyads; *M*_age_ = 20.83; 57.02% female; 112 Asian, 5 Black/African American, 37 Latino/a, 2 Native American, 56 White/Caucasian, 24 multiracial, 6 not listed).

### Design

Within each dyad, one participant was randomly assigned a regulator role, and the other participant was assigned a target role. To examine questions about extrinsic emotion regulation, we used multivariate linear regression models to determine the extent that independent variables from the regulator participants influenced the dependent variables from the target participants. A sensitivity analysis using G*Power indicated that for a two-predictor linear regression model, the present sample size of 121 dyads would be sufficient to detect a small effect size of *f*^2^ = 0.08 with 80% power.

We originally intended a between-subject design with three extrinsic prohedonic goal conditions for the regulator participants (i.e., upregulate positive emotions, downregulate negative emotions, and control). However, the manipulation of regulator goals was deemed to have failed because it did not influence regulators’ self-reported goals or their use of extrinsic emotion regulation strategies (see online supplemental material for manipulation details and relevant analyses). Given this, and because of the rich nature of the dataset, which involved real, ecologically valid extrinsic emotion regulation actions that participants self-generated to improve targets’ emotions alongside measurements from both dyadic members, we decided it was most appropriate to analyze the data based on participants’ natural variability in interpersonal emotion regulation actions collapsing across the originally intended manipulation.

## Data Availability

Data, code, and online supplemental materials are available on OSF at https://osf.io/kazep/.

## Procedure

### Overview (See Fig. 1)

**Fig. 1:**
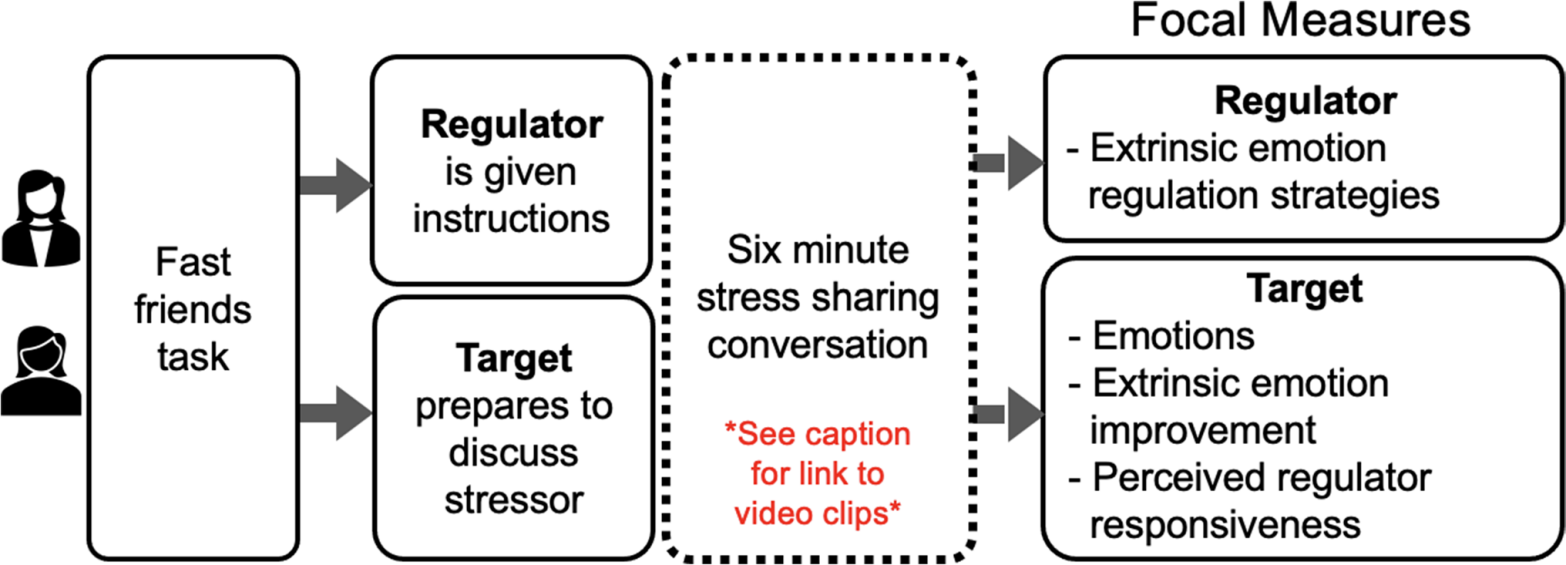
Overview of study procedure. View examples of these interactions at this link: https://osf.io/jmz69?view_only=fdac5950608a425aa273ba07ccd377ea

To get comfortable with the experimental environment and with each other, each pair of participants first interacted together in a 5-min fast friends task (Aron et al., 1997). Then, the participants were assigned either the role of the regulator or the target and given specific instructions depending on their role for the 6-min stress sharing conversation, a freeform interaction in which the target shared a stressful experience, and the regulator helped the target deal with that situation (see “Stress Sharing Conversation” section). At the end of the session, regulators rated their extrinsic strategy use. This measure was collected after the conversation to assess actual use not expected use of these extrinsic strategies. Targets rated their emotions, the extent that the regulator improved their emotions (i.e., extrinsic emotion improvement), and perceived regulator responsiveness. Other non-focal measures such as conversation intensity and sense of connection were also collected (see “Non-focal Measures” section) and used in analyses to account for alternative explanations.

Participants communicated with one another via the video conferencing platform, Zoom. To enhance the richness of and standardize the experimental environment, participants participated in a quiet and private location, turned off the self-view feature, and used full-screen mode. We used short and focused, candid conversations because they isolate the relationship between the regulators’ behavior and the targets’ outcomes while maintaining ecological validity. Many advantages exist for using remote video conferencing to study social interactions such as minimizing logistical issues and cost for the researchers and maximizing comfort and convenience for the participants. Video conferencing has become an incredibly common way to interact with others. For instance, Microsoft reported 270 million monthly active users of their video conferencing platform in January 2022 (Foley, 2022); thus, another advantage of this study is that it captures behavior that commonly occurs in the real world but is insufficiently examined. Furthermore, video conferencing effectively mirrors in-person interactions such that participants can see and speak to each other clearly and effortlessly. Thus, this tool does not forgo data quality. We would expect to see equivalent results in in-person interactions.

#### Fast Friends Task

The purpose of the 5-min fast friends task was for participants to get comfortable speaking about personal topics. Participants answered questions about themselves out loud with the other participant listening, alternating who answered first. Questions were selected from the closeness-generating questions developed by Aron et al. (1997) and included questions such as “Given the choice of anyone in the world, whom would you want as a dinner guest?” and “If you could wake up tomorrow having gained any one quality or ability, what would it be?” All dyads received the same questions in the same order.

#### Stress Sharing Conversation (See Tables 1 and 2)

The purpose of the 6-min stress sharing conversation was to generate a natural and emotional interaction between two participants in which we could examine extrinsic emotion regulation when targets’ are experiencing real and authentic emotions (see Table 1 for written examples of extrinsic strategies used during the stress sharing conversations and a link to a video of with example clips from the study). Table 2 provides a breakdown of the types of stressors discussed during the stress sharing conversation. The most common stressors included future uncertainty, school performance, interpersonal conflict, and health/COVID-19.

**Table 2:** Descriptive statistics for the conversations between participants

Mean depth of conversation: **3.65**
Mean meaningfulness of conversation: **4.18**
Percent of regulators who wanted to improve target emotions: **88%**
Distribution of topics of conversation
Future uncertainty (e.g., feeling lost, trouble finding a job, life transition issues): **25%**
School performance (e.g., poor grades, overwhelmed with coursework): **22%**
Interpersonal conflict (e.g., breakup, family dispute, strife among roommates): **16%**
Health or COVID-19 (e.g., managing an illness, fear of loved ones getting sick): **10%**
Financial (e.g., hospital bills, struggling to pay rent): **8%**
Loneliness (e.g., feeling isolated, lacking social support): **8%**
Balancing full-time employment (e.g., juggling school and work): **6%**
Other (e.g., grief, housing issues, transportation problems): **5%**

**Table 3:** Individual items for the extrinsic emotion regulation strategies

**Extrinsic reappraisal**
1. When I wanted the other person to feel less negative emotion (such as sadness or anger), I tried to help them change what they were thinking about.
2. When I wanted the other person to feel more positive emotion (such as joy or amusement), I tried to change what they were thinking about. 3. I influenced the other person’s emotions by suggesting alternative ways to think about the situation they are in
4. When the other person talked about their stressful situation, I helped them think about it in a way that calmed them down.
5. When I wanted the other person to feel less negative emotion, I tried to influence the way they were thinking about the situation.
6. When I wanted the other person to feel more positive emotion, I tried to change the way they were thinking about the situation.
**Extrinsic suppression**
1. I influenced the other person’s emotions by finding ways to keep them from expressing their emotions.
2. I acted in ways that made the other person keep their emotions to themselves.
3. When the other person was feeling positive emotions, I found ways to discourage them from expressing these emotions.
4. I managed the other person’s emotions by allowing them to feel and then release these emotions.
5. When the other person was feeling negative emotions, I tried to do things that kept them from expressing their emotions.
**Extrinsic acceptance**
1. I embraced the other person’s emotions, whatever they were.
2. I was comfortable with other person’s emotions.
3. I understood that the other person was going to have certain emotions at certain times and that was just fine.
4. I found it hard to come to terms with the other person’s emotions.
5. As far as influencing the other person’s emotions goes, I pretty much accepted the other person as they were.
6. Accepting the other person’s emotions was not easy for me.
7. I simply accepted the emotions of the other person as a natural response to the particular circumstances they are in.

For the stress sharing conversation, participants were randomly assigned to be the regulator or the target. In the instructions given to the participants, the regulator was referred to as the friend, and the target was referred to as the sharer to minimize any demand effects. Both participants were told that the role of the target is to speak about a stressor they are experiencing in their life right now, to discuss the details of why that experience happened, and to share how they feel. The stressor could include anything they perceive to be stressful at work, school, or home. Then, both participants were told that the role of the regulator is to talk with the target about that stressor. All regulators were given the goal to have a natural conversation with the target participants, and they were encouraged to share their thoughts and impressions, give advice, and ask questions. Participants who were not in the control condition were also told to either increase the positive emotions or decrease the negative emotions of the target participants. Compared to the control condition, these instructions did not successfully change regulators’ extrinsic prohedonic goals (*ps* > 0.10) nor did they significantly predict any of the outcome measures (*ps* > 0.20; see online supplemental materials for the complete instructions). After these instructions were given, the target was moved to a private Zoom breakout room for 5 min to brainstorm what they wanted to discuss during the stress sharing conversation.

## Focal Measures

### Regulator Extrinsic Strategy Use (See Table 3)

Regulators rated the following three extrinsic emotion regulation strategies that they used to manage the emotions of targets during the stress sharing conversation on 1 (strongly disagree) to 7 (strongly agree) scales: extrinsic reappraisal ($\mathrm{\alpha }$ = .77), extrinsic suppression ($\mathrm{\alpha }$ = .61), and extrinsic acceptance ($\mathrm{\alpha }$ = .80). The six-item extrinsic reappraisal and five-item extrinsic suppression scales were adapted from the cognitive reappraisal and the expressive suppression facets of the emotion regulation questionnaire (Gross & John, 2003) and from the trait-level interpersonal emotion regulation questionnaire (Gonzalez & John, 2021). The seven-item extrinsic acceptance scale was adapted from the trait-level interpersonal acceptance items of the interpersonal emotion regulation questionnaire (Gonzalez & John, 2021; see Table 3 for exact items).

### Target Emotion Improvement

#### Target Emotions

Targets rated the extent to which they felt positive and negative emotions during the stress sharing conversation using a measure adapted from a previous study on emotion regulation in social interactions (Impett et al., 2012). The five items for the positive emotion composite (α = .87) were “proud / good about yourself,” “compassionate / sympathetic / touched,” “grateful / appreciative / thankful,” “inspired / uplifted / elevated,” and “happy / pleased / joyful.” The six items for the negative emotion composite (α = .81) were “angry / irritable / frustrated,” “anxious / nervous,” “distressed / upset,” “guilty / embarrassed / ashamed,” “sad / down,” and “resentful / bitter / annoyed.” All items were measured on 1 (not at all) to 5 (very much) scales.

#### Extrinsic Emotion Improvement

Targets rated the extent to which regulators improved their emotions by upregulating their positive emotions and downregulating their negative emotions on 1 (strongly disagree) to 5 (strongly agree) scales (“The other participant succeeded in making me feel more positive emotions” and “The other participant succeeded in making me feel less negative emotions”). In figures and tables, these two items are referred to as extrinsic positive emotion upregulation and extrinsic negative emotion downregulation, respectively.

### Perceived Regulator Responsiveness

Targets rated the responsiveness of the regulators using adapted items from the Perceived Partner Responsiveness Scale (Reis et al., 2017) relevant to short conversations between strangers (e.g., “The other participant was responsive to my needs.”). Nine items were rated from 1 (strongly disagree) to 5 (strongly agree) by the target, and they assessed the caring, understanding, and validation shown by the regulator (α = .92).

## Non-focal Measures

To account for alternative variables that may improve target emotions during the stress sharing conversation, we collected measures on conversation intensity, sense of connection, regulator empathy, regulator goals/perceptions, and target trait affect.

### Conversation Intensity

Both regulator and target rated the meaningfulness and depth of the stress sharing conversation on 1 (strongly disagree) to 5 (strongly agree) scales.

### Sense of Connection

Both regulator and target rated their closeness and their motivation to affiliate at the end of the conversation. Closeness was measured using the Inclusion of Other in Self scale (Aron et al., 1992), and affiliation was measured using five items (Van Kleef et al., 2008) including “I would like to get to know the other participant better” and “I feel like the other participant and I are friends” on 1 (strongly agree) to 5 (strongly disagree) scale.

### Regulator Variables

#### Empathy

Before the start of the study, regulators rated their empathic concern and perspective-taking using the Interpersonal Reactivity Index (Davis, 1980) on 1 (does not describe me well) to 5 (describes me well) scales.

#### Regulator Extrinsic Prohedonic Goals

Regulators rated the extent they wanted to improve the targets’ positive and negative emotions during the conversation on 1 (strongly agree) to 5 (strongly disagree) scale.

#### Regulator Perception of Improved Target Emotion

Regulators rated to what extent they believed they successfully improved the targets’ positive and negative emotions on 1 (strongly disagree) to 5 (strongly agree) scales (“I succeeded in making the other participant feel more positive” and “I succeeded in making the other participant feel less negative”).

### Target Trait Affect

In order to account for targets’ emotions potentially influencing the regulators’ extrinsic strategy choices, at the beginning of the study, targets rated how much they felt positive and negative affect in general (in their daily life) on 1 (not at all) to 5 (very much) scales. The five items for the positive emotion composite (α = .74) were “proud / good about yourself,” “compassionate / sympathetic / touched,” “grateful / appreciative / thankful,” “inspired / uplifted / elevated,” and “happy / pleased / joyful.” The six items for the negative emotion composite (α = .85) were “angry / irritable / frustrated,” “anxious / nervous,” “distressed / upset,” “guilty / embarrassed / ashamed,” “sad / down,” and “resentful / bitter / annoyed.”

## Observer Ratings of Extrinsic Strategies

We obtained observer ratings of regulators’ strategy use from three trained behavioral coders. Behavioral coders watched videos of the entire conversation and rated both extrinsic reappraisal and extrinsic suppression on 1 (behavior is not present at all) to 5 (behavior is very frequently present) scales (see supplemental materials for instructions). We assessed inter-rater reliability on 13% of the data for extrinsic reappraisal (ICC = .75) and extrinsic suppression (ICC = .78). Six dyads were excluded due to improper recording or uploading of the video data.

Additionally, we attempted to obtain observer ratings of two forms of regulator extrinsic acceptance: regulators attempt at accepting the targets’ emotions (measured through self-report) and regulators attempt to lead the targets to accept their emotions (not measured through self-report). Due to low inter-rater reliability, we did not proceed with coding these variables.

## Results

### Regulator Extrinsic Strategy Use and Target Emotion Improvement

#### Extrinsic Reappraisal Predicted Target Emotion Improvement (See Fig. 2)

**Fig. 2:**
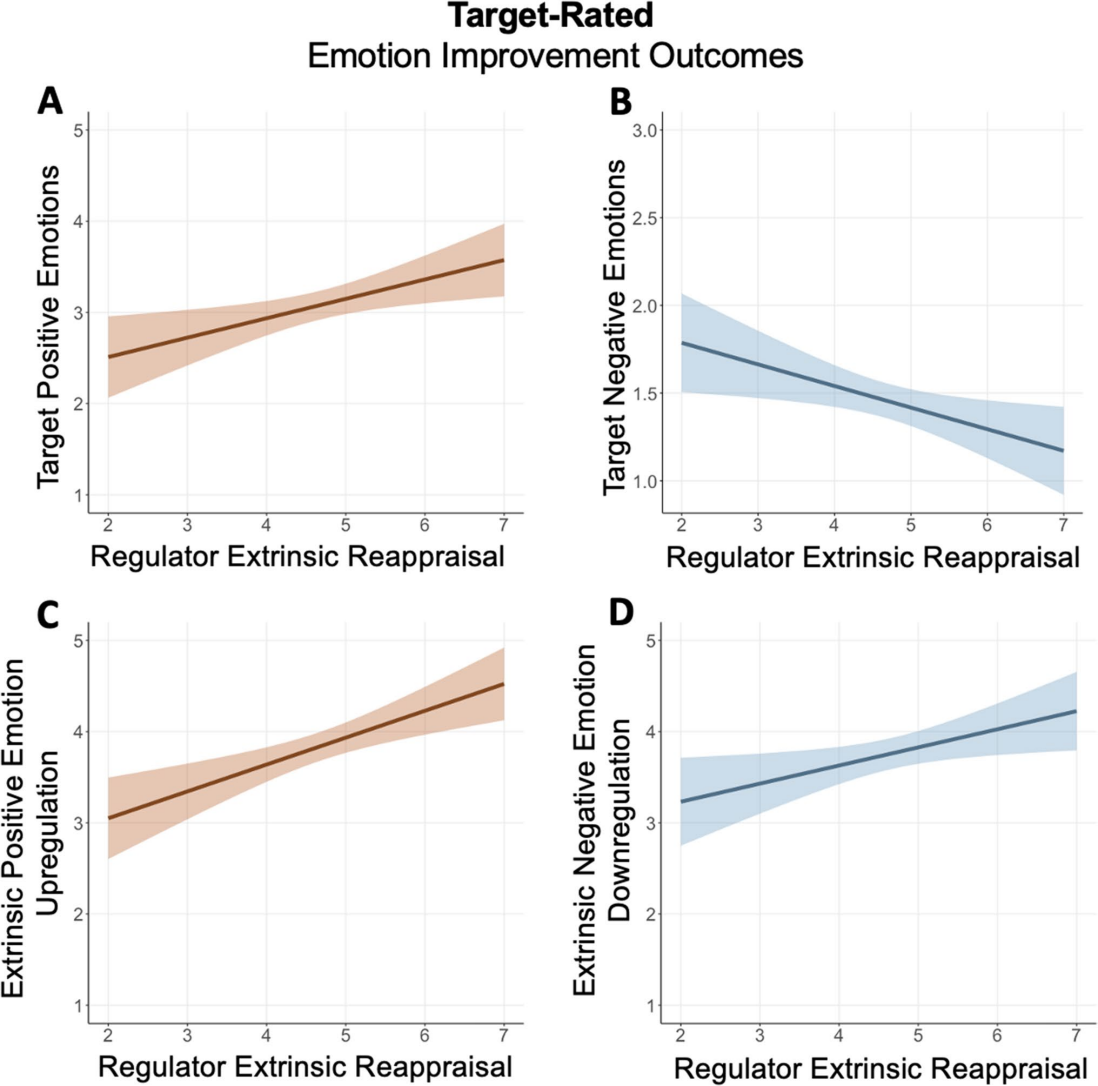
The influence of extrinsic reappraisal on target emotion improvement. When regulators used extrinsic reappraisal, targets experienced significantly elevated positive emotions (**A**) and significantly diminished negative emotions (**B**), and targets reported that regulators were responsible for their improved positive (**C**) and negative (**D**) emotions

First, we tested the relationship between regulator extrinsic reappraisal use and extrinsic emotion improvement using univariate linear regression models without control variables (for controls, see “Examining Alternative Explanations” section). When regulators used more extrinsic reappraisal during the stress sharing conversation, targets reported that regulators were significantly more successful at upregulating their positive emotions, *b* = 0.32, *t*(119) = 3.72, 95% CI [0.15, 0.49], *p* < 0.001, and at downregulating their negative emotions, *b* = 0.21, *t*(119) = 2.32, 95% CI [0.03 0.39], *p* = .022.

Then, we tested the relationship between regulator extrinsic reappraisal use and target emotions using univariate linear regression models without controls. When regulators used more extrinsic reappraisal, targets experienced significantly more positive emotion, *b* = 0.22, *t*(119) = 2.69, 95% CI [0.06, 0.37], *p* = .008, and less negative emotion, *b* =  − 0.12, *t*(119) =  − 2.47, 95% CI [− 0.22, − 0.02], *p* = .015, during the stress sharing conversation.

Additionally, we examined the alternative explanation for these results in the other causal direction—that regulators used more extrinsic reappraisal on targets with better affect (i.e., greater positive emotions and less negative emotions). We did not find any support for this alternative explanation. To examine this, we tested how target trait affects predicted regulator extrinsic reappraisal use. We found that targets with less positive affect at beginning of the study received significantly greater extrinsic reappraisal from regulators, *b* =  − 0.32, *t*(117) =  − 2.04, 95% CI [− 0.62, − 0.01], *p* = .044. Target trait negative affect was not significantly associated with regulator extrinsic reappraisal use, *b* =  − 0.08, *t*(117) =  − 0.62, 95% CI [− 0.32, 0.17], *p* = .54. This suggests that regulators used more extrinsic reappraisal on those who entered the study in a worse affective state and, on average, successfully uplifted them during the conversation. Finally, we controlled for target trait affect in our main analyses. When we did, we still found that when regulators used more extrinsic reappraisal, targets experienced significantly more positive emotion, *b* = 0.27, *t*(117) = 3.45, 95% CI [0.11, 0.42], *p* < .001, and less negative emotion, *b* = 0.12, *t*(117) =  − 2.49, 95% CI [− 0.22, − 0.02], *p* = .014, indicating that targets’ affect at the start of the study could not explain the main results.

#### Extrinsic Suppression and Acceptance Did Not Predict Target Emotion Improvement

Neither extrinsic suppression nor extrinsic acceptance was significantly associated with targets’ emotions or extrinsic emotion improvement (see Table 4).

**Table 4:** Means, standard deviations, and correlations of focal measures

	*M*	*SD*	1	2	3	4	5	6	7
Regulator-rated variables									
1. Extrinsic reappraisal	4.67	1.01							
2. Extrinsic acceptance	5.97	0.75	− .05						
3. Extrinsic suppression	2.50	0.88	.11	− .56**					
Target-rated variables									
4. Positive emotions	3.08	0.90	.24**	.11	− .06				
5. Negative emotions	1.46	0.56	− .22*	− .07	− .03	− .35**			
6. Extrinsic positive emotion upregulation	3.83	0.93	.32**	.09	.03	.61**	− .27**		
7. Extrinsic negative emotion downregulation	3.76	0.97	.21*	.15	.00	.50**	− .23*	.61**	
8. Perceived regulator responsiveness	4.10	0.65	.20*	.30**	− .22*	.64**	− .22*	.41**	.46**

Extrinsic positive emotion upregulation is the extent that the target perceived the regulator to successfully upregulate their positive emotions; extrinsic negative emotion downregulation is the extent that the target perceived the regulator to successfully downregulate their negative emotions

*p < .05

**p < .01

## The Role of Responsiveness in Target Emotion Improvement

### Responsiveness Predicted Target Emotion Improvement

When targets perceived greater regulator responsiveness, they experienced significantly greater positive emotion, *b* = 0.89, *t*(119) = 9.08, 95% CI [0.70, 1.09], *p* < .001, and lower negative emotion, *b* =  − 0.20, *t*(119) =  − 2.51, 95% CI [− 0.35, − 0.04], *p* = .013. When targets perceived greater regulator responsiveness, they also reported that regulators were significantly more successful at upregulating their positive emotions, *b* = 0.63, *t*(119) = 4.85, 95% CI [0.37, 0.89], *p* < .001, and at downregulating their negative emotions, *b* = 0.71, *t*(119) = 5.64, 95% CI [0.46, 0.96], *p* < .001.

### Extrinsic Strategy Use Predicted Greater Responsiveness

Targets perceived greater regulator responsiveness when regulators used more extrinsic reappraisal, *b* = 0.13, *t*(119) = 2.24, 95% CI [0.02, 0.24], *p* = .027, and more extrinsic acceptance, b = 0.20, *t*(119) = 3.47, 95% CI [0.08, 0.31], *p* < .001, whereas targets perceived less regulator responsiveness when regulators used more extrinsic suppression, *b* =  − 0.14, *t*(119) =  − 2.44, 95% CI [− 0.26, − 0.03], *p* = .01 (see Table S1 the correlations between specific facets of responsiveness and extrinsic strategies).

### Responsiveness as a Mediator of Target Emotion Improvement (See Fig. 3)

**Fig. 3:**
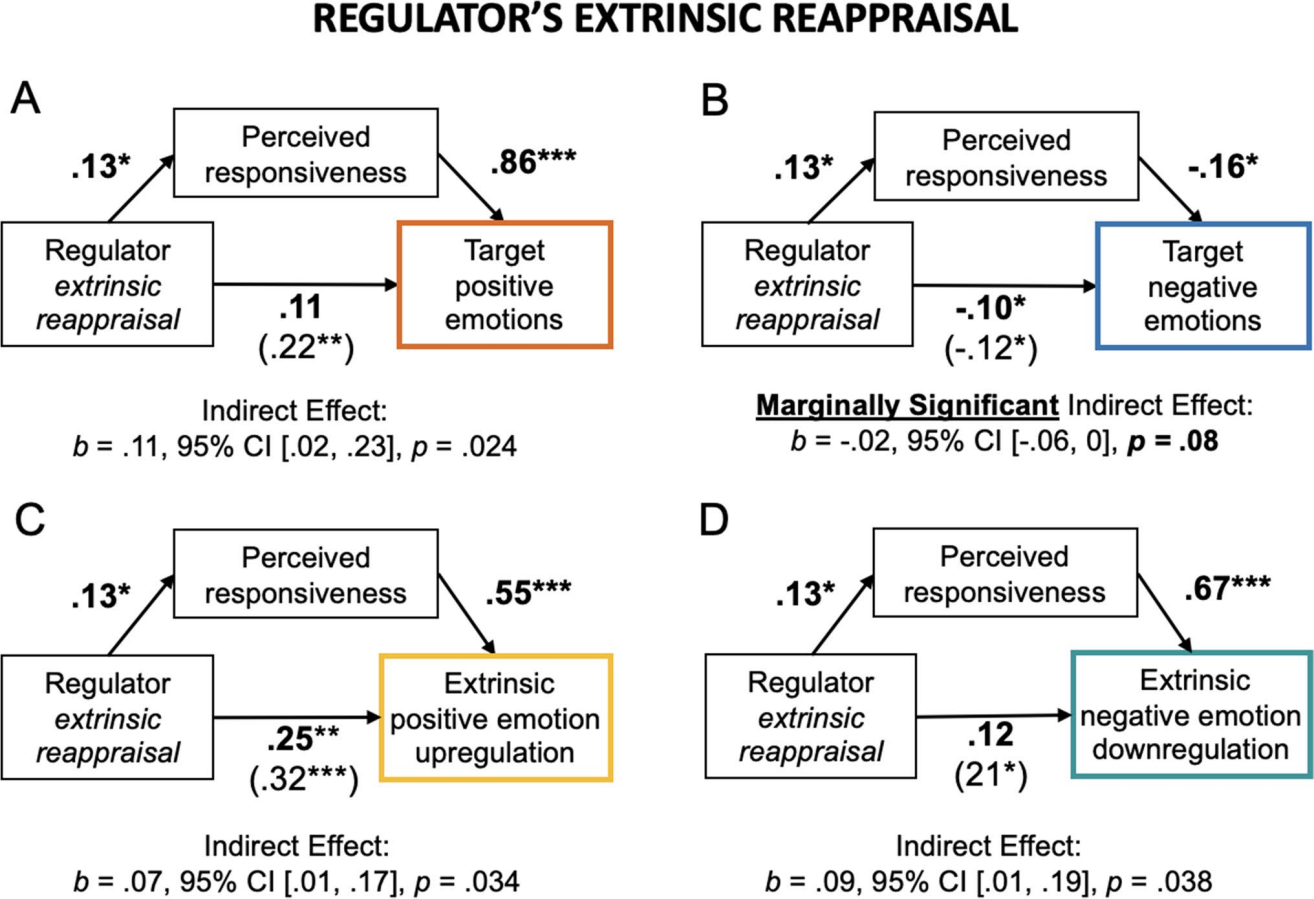
Greater perceived regulator responsiveness significantly mediated the relationship between extrinsic reappraisal and target positive emotion (A), regulator success at upregulating target positive emotion (C), and regulator success at downregulating target negative emotion (D). Greater perceived regulator responsiveness marginally mediated the relationship between extrinsic reappraisal and target negative emotion (B). *p < .05, **p < .01, ***p < .001

To examine the extent that perceived regulator responsiveness mediated the relationship between regulator extrinsic strategies and target emotion improvement, we ran multiple mediation models using the mediation R package (Tingley et al., 2014). We tested the significance of the indirect effects using 1,000 bootstrapped samples. Figure 3 illustrates and reports the indirect effects of greater perceived regulator responsiveness on target emotion improvement, measured by the four outcomes variables (i.e., target positive emotion, target negative emotion, regulator success at upregulating target positive emotion, and regulator success at downregulating target negative emotion). These mediation models suggest that regulators who used more extrinsic reappraisal improved target emotions, partially by being perceived as a more responsive interaction partner.

**Fig. 4:**
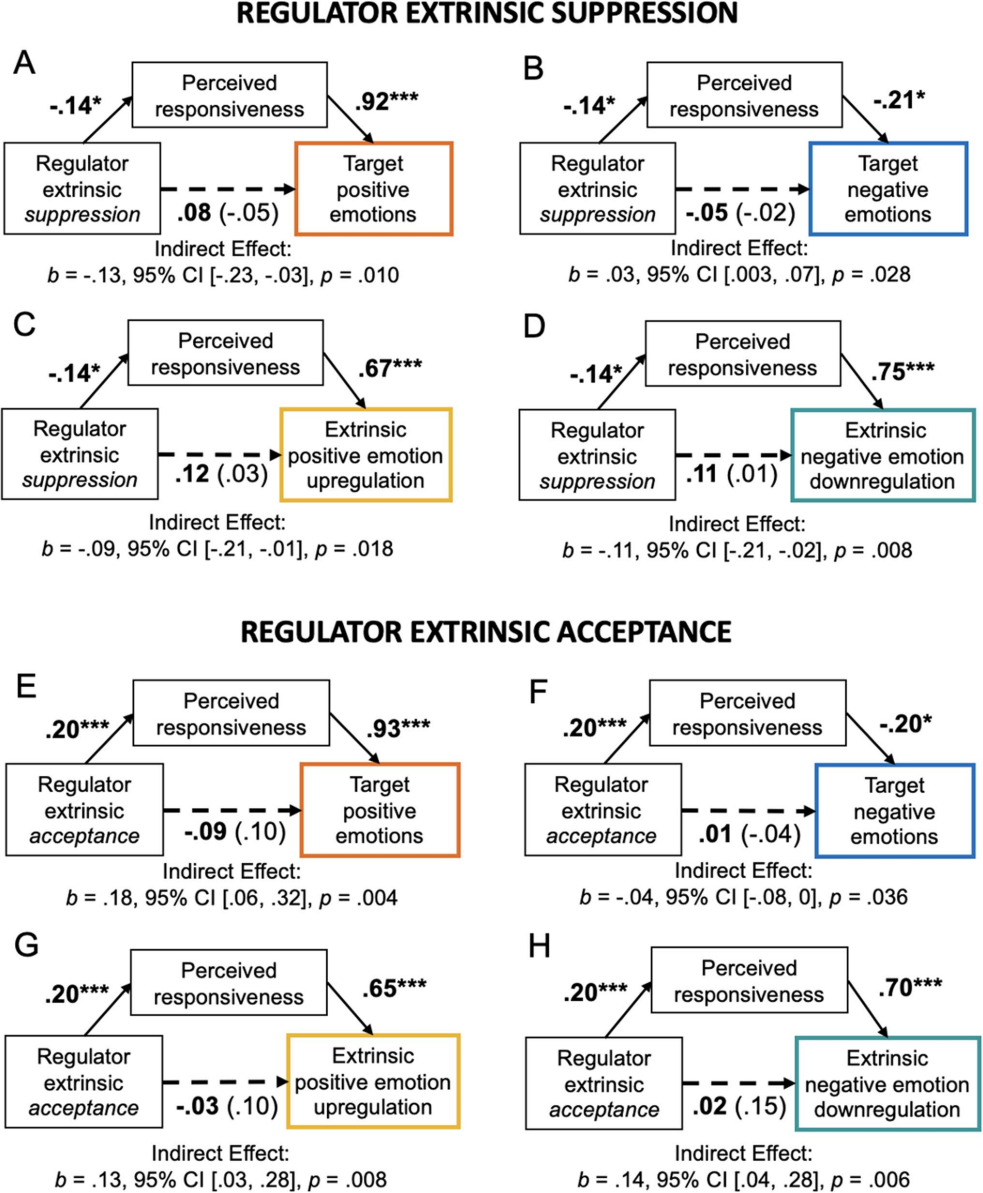
The mediating role of perceived regulator responsiveness on target emotion improvement for extrinsic suppression and extrinsic acceptance*.* Lower perceived regulator responsiveness fully mediated the relationship between greater extrinsic suppression and worsened target emotion (**A**, **B**) and between greater extrinsic suppression and the unsuccessful improvement of target emotion (**C**, **D**). Greater perceived regulator responsiveness fully mediated the relationship between greater extrinsic acceptance and better target emotion (**E**, **F**) and between greater extrinsic acceptance and the successful improvement of target emotion (**G**, **H**). Dashed lines refer to non-significant relationships. *p < .05, **p < .01, ***p < .001

Although extrinsic suppression and extrinsic acceptance were not significantly associated with target emotion improvement, these two extrinsic strategies were significantly associated with perceived regulator responsiveness. Therefore, we examined the mediating role of perceived regulator responsiveness on the relationship between these two strategies and target emotion improvement. In all models (see Fig. 4), perceived regulator responsiveness fully mediated the relationship between the extrinsic strategies and target emotion improvement. In summary, these results suggest that extrinsic acceptance was effective at improving target emotion due to greater perceived regulator responsiveness, and that extrinsic suppression was not effective at improving target emotion due to diminished perceived regulator responsiveness.

## Examining Alternative Explanations

Due to the cross-sectional nature of our data, we tested multiple alternative explanations that could account for an independent increase in both regulator extrinsic reappraisal use and target emotions. First, we expected that conversation intensity (i.e., meaningfulness and depth), sense of connection (i.e., closeness and affiliation), and regulator empathy (i.e., empathic concern and perspective-taking; see Table S2 for descriptive statistics) would be associated with target emotions. Thus, we controlled for these variables in linear regression models to predict target emotions, and we found that regulator extrinsic reappraisal use remained a significant predictor of target emotions (see Table 5). Our findings suggest that regulator extrinsic reappraisal use is a critical predictor of improved target emotions irrespective of conversation intensity, sense of connection, and regulator empathy. Furthermore, extrinsic reappraisal did not significantly correlate with conversation intensity, sense of connection, or regulator empathy (see Table S3) which indicates that these variables did not mediate the effectiveness of extrinsic reappraisal.

**Table 5:** Regressions predicting target emotions from extrinsic reappraisal with control variables

	Target negative emotion				Target positive emotion			
Predictors	B	SE	*t*	*p*	B	SE	*t*	*p*
Conversation intensity								
Meaningfulness (*t*)	− 0.05	0.46	− 0.78	.43	0.18	0.08	2.40	.02*
Meaningfulness (*r*)	− 0.04	0.07	− 0.50	.62	− 0.17	0.10	− 1.71	.09
Depth (*t*)	0.09	0.88	1.66	.10	0.05	0.06	0.85	.40
Depth (*r*)	0.10	0.05	1.58	.12	− 0.17	0.07	− 2.24	.03*
Sense of connection								
Closeness (*t*)	− 0.01	0.06	− 0.25	.80	0.25	0.06	4.35	.00***
Closeness (*r*)	0.01	0.05	0.21	.83	0.02	0.06	0.43	.67
Affiliation (*t*)	− 0.14	0.08	− 1.67	.10	0.30	0.09	3.20	.001**
Affiliation (*r*)	− 0.11	0.09	− 1.21	.23	0.01	0.11	0.11	.91
Regulator empathy								
Empathic concern	− 0.00	0.11	− 0.00	1.00	0.18	0.12	1.48	.14
Perspective taking	− 0.04	0.11	− 0.41	.69	0.19	0.12	1.50	.14
Extrinsic reappraisal	− 0.15	0.05	− 2.76	.01**	0.14	0.06	2.23	.03*
Fit	*F*	*df*	*p*	*R*^2^	*F*	*df*	*p*	*R*^2^
	2.03	11,105	.03*	.09	13.26	11,105	.00***	.05
Multicollinearity	VIFs < 1.94				VIFs < 1.94			

*t*, target-rated; *r*, regulator-rated

*p < .05, **p < .01, ***p < .001

Another alternative explanation is that, at the end of the conversation, regulators were motivated to endorse greater extrinsic reappraisal use when they perceived that they improved target emotions. We controlled for this motivational bias by asking regulators to what extent they perceived that they improved target emotions at the end of the conversation. We found that when we included this in predicting target emotions, regulator extrinsic reappraisal use remained significant, and there was no significant interaction (see Table S4). In other words, regulator extrinsic reappraisal use was positively associated with better target emotions whether or not regulators perceived that they improved target emotions.

A final alternative explanation is that a regulator who used extrinsic reappraisal also held the extrinsic prohedonic goal to improve the target’s emotions, and it was this goal that drove other behaviors which resulted in improved target emotions (see Tamir et al., 2019 for the distinction between goals and strategies in emotion regulation). In this study, 88% of regulators had the goal to improve target emotions, and having this goal positively correlated with greater extrinsic reappraisal use and extrinsic acceptance use but negatively correlated with greater extrinsic suppression use (see online supplemental). When we included regulators’ extrinsic prohedonic goal in the linear regression models to predict target emotions, we found that this goal was not significantly associated with target emotions and did not significantly interact with regulator extrinsic reappraisal use, and regulator extrinsic reappraisal use remained significant (see Table S5). In summary, the positive relationship between extrinsic reappraisal use and target emotions was not confounded by regulators’ goals.

## Observer Ratings of Extrinsic Reappraisal and Suppression

Observer ratings of both regulator extrinsic reappraisal use (*M* = 2.81, SD = 1.41) and regulator extrinsic suppression use (*M* = 1.99, SD = 1.16) positively correlated with regulators’ self-ratings of these strategies, respectively (*r*(113) = .40, *p* < .001 and *r*(113) = 0.38, *p* < .001). Furthermore, we obtained similar results when using observer ratings to predict emotion outcomes and perceived partner responsiveness (see Table S10) such that observer-rated extrinsic reappraisal was positively associated with better target emotion outcomes and greater perceived regulator responsiveness and that observer-rated extrinsic suppression was not significantly associated with target emotion outcomes but was negatively associated with perceived regulator responsiveness.

## Discussion

In the present study, we examined the effectiveness of three extrinsic strategies in candid dyadic conversations. These strategies are exhaustively studied in the intrapersonal context. However, whether these strategies would show similar consequences in an interpersonal setting is a pressing question. We demonstrated that extrinsic emotion improvement can successfully happen in short conversations between newly acquainted people without receiving any specific instruction on how to regulate the emotions of others. We found that greater use of extrinsic reappraisal was directly associated with improved target emotions, whereas extrinsic suppression and acceptance were not. Emotion improvement was measured by asking target participants about their emotions and about the regulators’ success at improving their emotions. We found convergent findings on all four distinct measures in our focal analyses.

Although extrinsic suppression and acceptance had no direct association with improved target emotions, all extrinsic strategies had an indirect association with improved target emotions through perceived regulator responsiveness. As predicted, responsiveness mediated improved target emotions such that extrinsic acceptance and reappraisal enhanced responsiveness, whereas extrinsic suppression diminished responsiveness. It is possible that the effectiveness of these strategies could also be moderated by responsiveness; however, our data does not suggest this (see Table S6). Instead, we found that these extrinsic actions were associated with distinct social perceptions which impacted their relationship to improving others’ emotions. Finally, we found that observer-ratings of extrinsic reappraisal and extrinsic suppression corresponded with regulator self-ratings and that there was a similar pattern of results on target emotion outcomes.

## Limitations and Future Directions

Some limitations should be considered when interpreting these findings. First, this study is correlational in nature; while the present findings fit within the causal framework of our theorizing and the observational data provides additional support for our hypotheses, the present results do not speak directly to causality. It is possible that the regulators’ use of extrinsic reappraisal is in response to the way in which targets disclose their distress. Therefore, future research should manipulate extrinsic reappraisal to determine its effectiveness.

Second, it is important to note that not all regulator participants had the same (if any) interpersonal goals. Thus, attempts at changing targets’ emotions may have been conversational-directed depending on the targets’ disclosure instead of goal-directed. This raises the question—what trigger regulators to have these goals? In future research, it would be useful to examine whether regulators set these goals because of their own motivations or because of the type of disclosure by the target. For example, a target person may seek emotional support but receive problem-oriented support (Liu et al., 2021) because of the way they present their distress. In other words, the regulator’s goals may be shaped by the target (i.e., non-deliberate intrinsic emotion regulation).

Finally, although we found that extrinsic reappraisal was directly associated with improved target emotions, we do not know which specific reappraisal tactics were most effective. Reappraisal can work both by changing the construal of the situation or by changing the goals for the situation (Uusberg et al., 2019), and one of these reappraisal tactics may work better than the other in the interpersonal context. Furthermore, these reappraisal tactics could interact with intrinsic interpersonal emotion regulation goals (i.e., when an individual desires to change their own emotions through the help of others). Often, people seek out others for emotional help (Campos et al., 2011; Williams et al., 2018). Yet, people may avoid individuals who challenge their thinking regardless of whether it could help them in the long run (Behfar et al., 2020). This mismatch between the extrinsic tactics that the regulator uses and what the target wants may impact the effectiveness of extrinsic reappraisal. In future research, it will be important to identify which types of extrinsic reappraisal people seek out or avoid.

In this study, extrinsic acceptance was measured by asking regulators if they accept the emotions of the target. Although we conceptually believe that this may lead to regulators helping targets accept their emotions, this may not necessarily be the case. In future research, it is critical to examine if this link exists between regulators’ acceptance of targets’ emotions and their attempts to aid targets in accepting their emotions. These attempts at helping a target accept their emotions are possibly an effective interpersonal emotion regulation strategy which was not directly measured in this study.

Future research should compare the emotional and social consequences of other interpersonal strategies such as advice-giving, humor, and affection (Niven et al., 2009). These strategies may work better than extrinsic reappraisal depending on the situation. For instance, extrinsic reappraisal may help someone deal with rejection, whereas advice-giving may work better at helping someone manage the emotions involved in social conflict. An important factor to consider in future research is the controllability of the situation (Ford & Troy, 2019) and if extrinsic reappraisal works better for situations with low controllability (e.g., grief) like its intrapersonal counterpart.

## Supplementary Information

Below is the link to the electronic supplementary material.

Supplementary file1 (DOCX 428 KB)

## Additional Information

### Data Availability

The experiment reported in this article was not formally preregistered. Data, code, and online supplemental materials are available on OSF at https://osf.io/kazep/, and a preprint is available at https://psyarxiv.com/ejdth.
