# Supplementary material for: How to Improve Others’ Emotions: Reappraise and be Responsive
Source: Affect Sci. 2023 Apr 15;4(2):233–47. doi: 10.1007/s42761-023-00183-4 (PMC10104771; doi:10.1007/s42761-023-00183-4)
Supplement: Supplementary file 1 — Supplementary file1 (DOCX 428 KB) [file 42761_2023_183_MOESM1_ESM.docx]

**Supplemental Materials**

**Methods**

***Regulator Instructions***

The following italicized paragraph is the complete instructions given privately to the regulator by the experimenter. The text in parentheses refer to the three manipulated goal conditions separated by slashes in following order: control, upregulate target’s positive emotions, or downregulate target’s negative emotions. Regulators were randomly assigned to one condition.

*In this study, you were randomly assigned to be the friend and your partner the sharer. Both of you will have a 6-minute conversation in which your partner will share with you a stressor that they are dealing with. During this conversation, it’s your goal to have (****a natural conversation / a natural conversation that increases the positive feelings / a natural conversation that decreases the negative feelings****) of the sharer.*

*Your partner will share with you the stressor they are experiencing. During the conversation it’s your job to (****just have a natural conversation / make them feel more positive feelings / make them feel less negative feelings****). Research shows that the best way to have these tough conversations is to (****be natural / increase their positive feelings / decrease their negative feelings****). There are many ways to do this when you talk with someone. You can share your thoughts and impressions. You can ask them questions and listen to what they have to say. You can also tell them what you would do in this situation. Depending on how the conversation goes and what they share, there are many ways you can interact with your partner, but your goal throughout the entire conversation is to (****have a natural conversation / make them feel more positive feelings / make them feel less negative feelings****). This is open-ended, so both of you are welcome to speak about anything you think is related to the stressor such as your own thoughts, feelings, impressions, beliefs and stories.*

*In summary, you and your partner will discuss a stressful event they are experiencing. During this conversation, your goal is to (****have a natural conversation / increase their positive feelings / decrease their negative feelings****).*

**Observer-rating Instructions**

For each video, score the strategies on a 1 (*behavior is not present at all*) to 5 (*behavior is very frequently present*) scale. The behavior of extrinsic reappraisal is any attempts to try to change how someone else thinks about a negative situation. Some examples of how to do this are that a regulator can help the other person reframe the situation to see it in a positive way, attempt to change the other person’s beliefs about the situation, help the other person reinterpret what the situation was or what their emotions mean, and the regulator may attempt to change the targets’ goals/expectations related to the situation. The behavior of extrinsic suppression is any attempts to try to directly or indirectly suppress the emotions of the other person. Some examples of how to do this include changing the subject, ignoring the other person’s emotions, and reacting in a way that disregards the other person’s emotional expression.

***Measures***

**Target’s Intrapersonal Strategies.** Emotion regulation strategies employed by targets to manage their own emotions during the stress sharing task were measured on 1 (*strongly disagree*) to 7 (*strongly agree*) scales from the cognitive reappraisal and the expressive suppression facets of the emotion regulation questionnaire (Gross & John, 2003).

**Results**

***Manipulation did not Influence Goals, Extrinsic Strategies or Regulation Success***

The instructions provided to the regulators did not successful change their extrinsic hedonic goals (ps > .10) nor did they significantly predict any of the outcome measures (ps > .20).

***Extrinsic Suppression and Acceptance Did Not Influence Regulation Success***

**Extrinsic Suppression.** The regulator’s *suppression use* did not significantly predict the targets’ positive emotions *b* = -.05, *t*(119) = -.63, 95% CI [-.21, .11], *p* = .53 or negative emotions *b* = -.02, *t*(119) = -.34, 95% CI [-.12, .08], *p* = .73.

The regulators’ *suppression use* did not significantly predict success at upregulating the targets’ positive emotions *b* = .03, *t*(119) = .28, 95% CI [-.16, .21], *p* = .78 or success at downregulating the targets’ negative emotions *b* = .0002, *t*(119) = .002, 95% CI [-.18, .18], *p* = .99.

**Extrinsic Acceptance.** The regulators’ *acceptance use* did not significantly predict the targets’ positive emotions *b* = .10, *t*(119) = 1.18, 95% CI [-.07, .26], *p* = .24 or negative emotions *b* = -.04, *t*(119) = -.72, 95% CI [-.14, .07], *p* = .48.

The regulators’ *acceptance use* did not significantly predict success at upregulating the targets’ positive emotions *b* = .09, *t*(119) = 1.01, 95% CI [-.09, .27], *p* = .31 or success at downregulating the targets’ negative emotions *b* = .15, *t*(119) = 1.69, 95% CI [-.03, .33], *p* = .09.

***Did Regulator Goals Influence the Success of Extrinsic Emotion Regulation?(see Figure 1)***

The regulators’ goal to upregulate the positive emotions of the target did significantly predict successful upregulation of the targets’ positive emotions rated by the target, *b* = .19,*t*(119) = 2.06, 95% CI [.007, .36], *p* = .041, but did not significantly predict positive emotions *b* = .01, *t*(119) = 0.15, 95% CI [-.15, .18], p = .88, negative emotions *b* = -.01, *t*(119) = -0.20, 95% CI [-.11, .09], *p* = .84, and the downregulation of the targets’ negative emotions *b* = .08, *t*(119) = 0.83, 95% CI [-.11, .26], *p* = .41.

The regulators’ goal to downregulate the targets’ negative emotions did significantly predict successful downregulation of the targets’ negative emotions rated by the target, *b* = .21, *t*(119) = 2.40, 95% CI [.04, .39], *p* = .018, but did not significantly predict positive emotions *b* = .08,*t*(119) = 0.94, 95% CI [-.08, .24], *p* = .34, negative emotions *b* = .06, *t*(119) = 1.09, 95% CI [-.05, .16], *p* = .28, and the upregulation of the targets’ positive emotions *b* = .09,*t*(119) = 0.97, 95% CI [-.09, .27], *p* = .33.


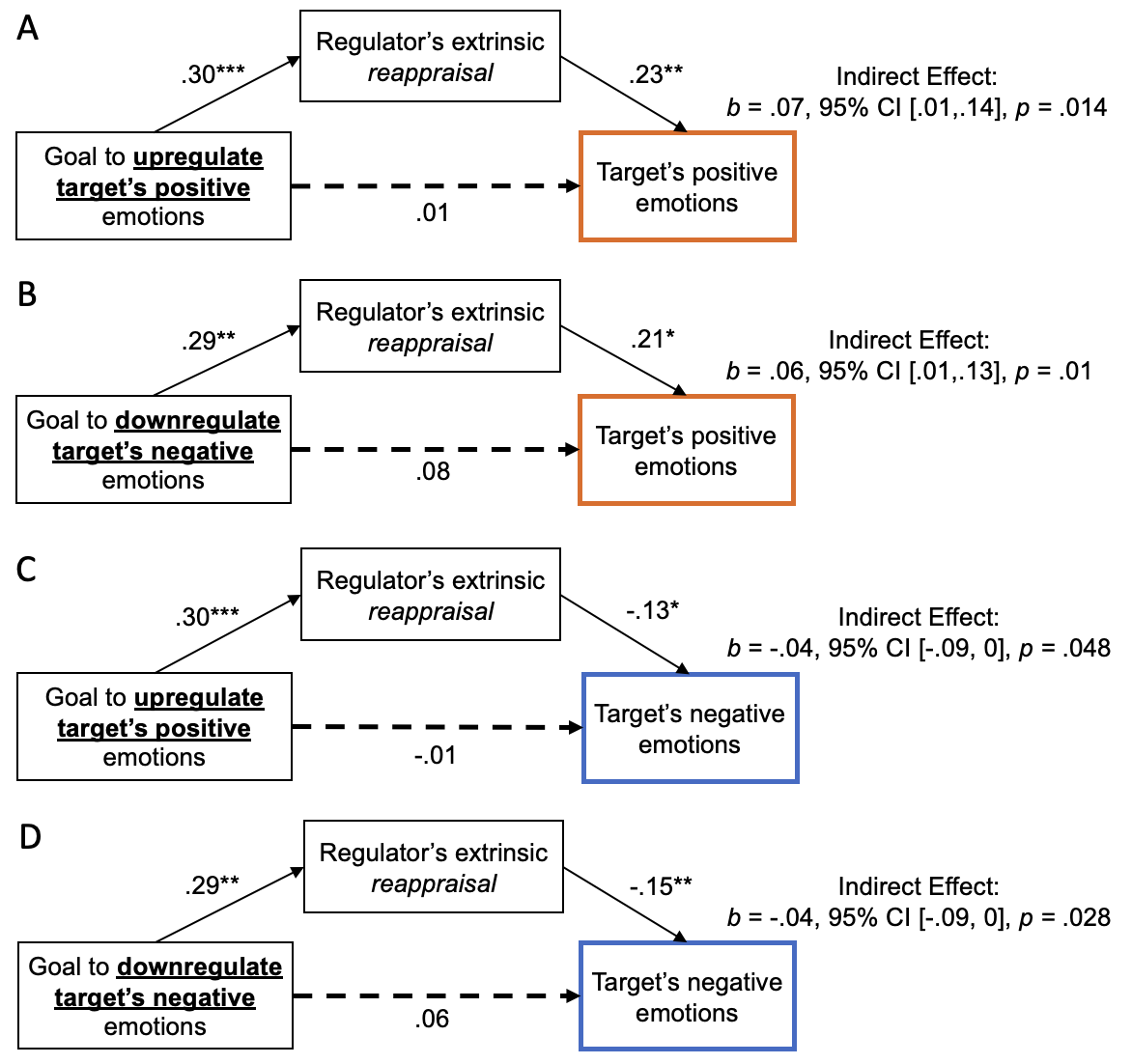


***Figure 1.*** *Extrinsic hedonic goals influence targets’ emotions indirectly when mediated by regulators use of extrinsic reappraisal.* When mediated by extrinsic reappraisal, greater extrinsic hedonic goals indirectly resulted in elevated positive emotions of the target (panel A & B) and resulted in diminished negative emotions of the target (panel C & D). Dashed lines refer to non-significant relationships.

* *p* < .05, ** *p* < .01, *** *p* < .001

***How did Intrapersonal Strategies Influence Emotions?***

We examined the extent that *intr*apersonal strategies influenced the emotions of the target. Reappraisal use by the target did not significantly predict their positive emotions, *b* = .03, *t*(119) = .35, 95% CI [-.13, .19], *p* = .72 or their negative emotions, *b* = -.02, *t*(119) = -.03, 95% CI [-.13, .07], *p* = .56. When the targets used suppression, they experienced significantly less positive emotions, *b* = -.23, *t*(119) = -2.84, 95% CI [-.39, -.07], *p* = .005, and they experienced marginally more negative emotions *b* = .10, *t*(119) = 1.90, 95% CI [-.004, .20], *p* = .06.

**Regulator Goals Were Associated with Strategy Use and Success (see Figure 2)**

The regulators’ goal to upregulate the targets’ positive emotions and to downregulate the targets’ negative emotions were positively correlated with each other, *r*(119) = 0.56, *p* < .001.

***Greater Extrinsic Prohedonic Goals Predicted More Extrinsic Reappraisal Use***

Both extrinsic prohedonic goals (upregulate positive: *b* = .30, *t*(119) = 3.48, 95% CI [.13, .48], *p* < .001; downregulate negative: *b* = .29, *t*(119) = 3.29, 95% CI [.11, .46], *p* = .001) predicted greater use of extrinsic reappraisal. When both goals were included in the same regression model, only the goal to upregulate targets’ positive emotions predicted greater use of extrinsic reappraisal (upregulate positive: *b* = .21, *t*(118) = 1.65, 95% CI [.0003, .41], *p* = .050, downregulate negative, *b* = .17, *t*(118) = 1.65, 95% CI [-.03, .38], *p*= .10).

We examined the indirect influence of regulator goals on targets’ emotions when regulators used extrinsic reappraisal. We tested the significance of the indirect effects using 1000 bootstrapped samples through the mediation R package (Tingley et al., 2014). Both goals did indirectly improve the targets’ emotions through the regulators’ use of extrinsic reappraisal.

***Greater Extrinsic Hedonic Goals Predicted Less Extrinsic Suppression Use***

When regulators had the goal to downregulate the targets’ negative emotions, they used significantly less extrinsic suppression, *b* = -.18, *t*(119) = -2.02, 95% CI [-.36, -.004], *p* = .045. The goal to upregulate the targets’ positive emotions did not significantly predict extrinsic suppression, *b* = -.11, *t*(119) = -1.19, 95% CI [-.29, .07], *p*= .24.

***Greater Extrinsic Hedonic Goals Predicted More Extrinsic Acceptance Use***

When regulators had the goal to downregulate the targets’ negative emotions, they used significantly more extrinsic acceptance, *b* = .23, *t*(119) = 2.62, 95% CI [.06, .41], *p* = .010. The goal to upregulate the targets’ positive emotions marginally predicted extrinsic acceptance, *b* = .16, *t*(119) = -1.79, 95% CI [-.02, .34], *p* = .08.


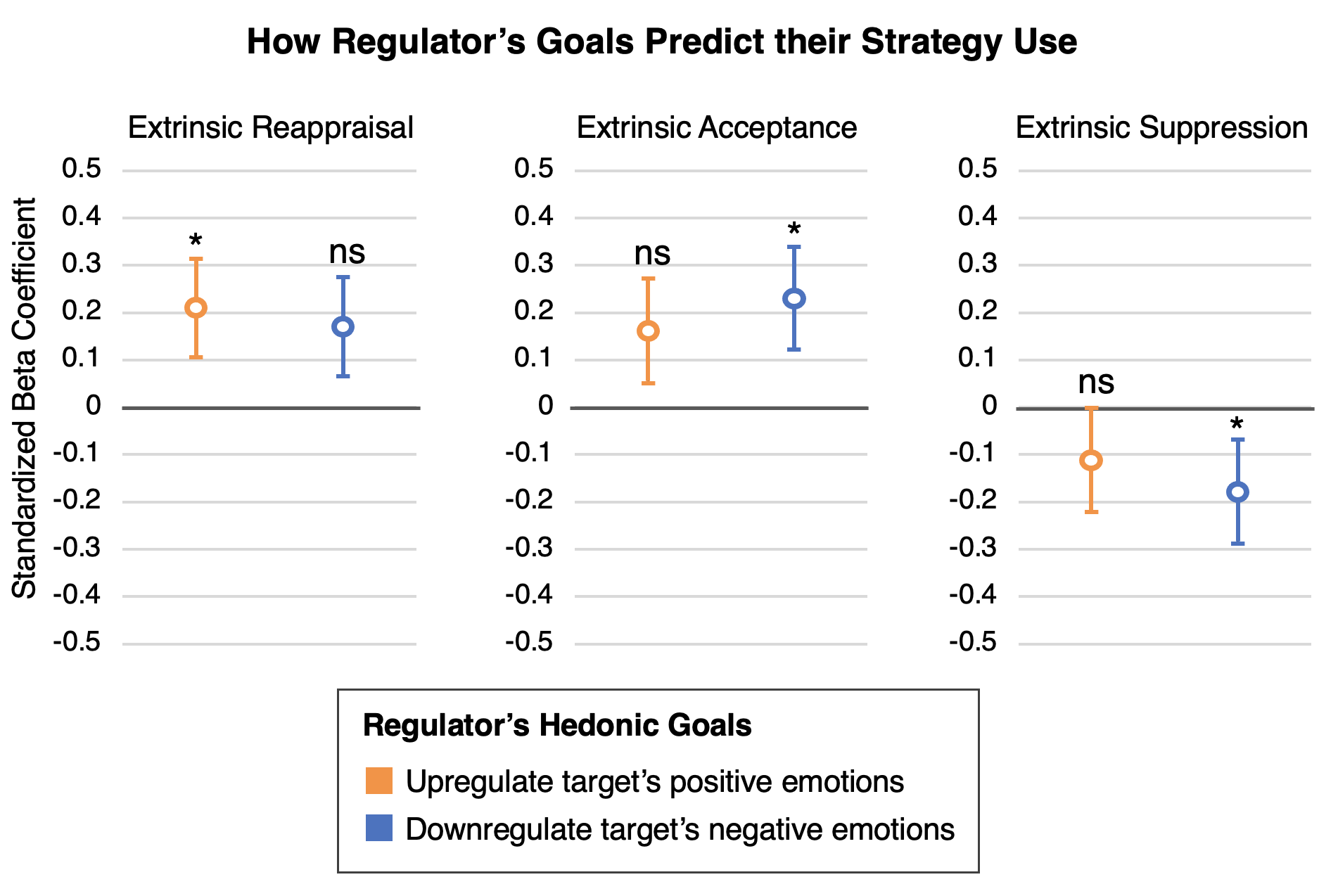


***Figure 2.*** *The differential influence of goals on strategy use.* The goal to upregulate the target’s positive emotions significantly predicted greater extrinsic reappraisal use, whereas the goal to downregulate the target’s negative emotions significantly predicted greater extrinsic acceptance use and less extrinsic suppression use.

* *p* < .05, ns = non-significant.

Table 1.

*Extrinsic Strategies and Perceived Regulator Responsiveness Facets*

| Variable | *M* | *SD* | 1 | 2 | 3 | 4 | 5 |
| --- | --- | --- | --- | --- | --- | --- | --- |
|  |  |  |  |  |  |  |  |
| 1. Extrinsic Reappraisal | 4.67 | 1.01 |  |  |  |  |  |
|  |  |  |  |  |  |  |  |
| 2. Extrinsic Acceptance | 5.97 | 0.75 | -.05 |  |  |  |  |
|  |  |  | [-.23, .13] |  |  |  |  |
|  |  |  |  |  |  |  |  |
| 3. Extrinsic Suppression | 2.50 | 0.88 | .11 | -.56** |  |  |  |
|  |  |  | [-.07, .29] | [-.67, -.43] |  |  |  |
|  |  |  |  |  |  |  |  |
| 4. Validation | 4.23 | 0.69 | .21* | .22* | -.14 |  |  |
|  |  |  | [.03, .37] | [.05, .39] | [-.31, .04] |  |  |
|  |  |  |  |  |  |  |  |
| 5. Understanding | 3.90 | 0.74 | .21* | .21* | -.13 | .78** |  |
|  |  |  | [.03, .37] | [.03, .38] | [-.30, .05] | [.70, .84] |  |
|  |  |  |  |  |  |  |  |
| 6. Concern | 4.17 | 0.70 | .13 | .39** | -.33** | .72** | .70** |
|  |  |  | [-.05, .30] | [.23, .53] | [-.48, -.16] | [.63, .80] | [.59, .78] |
|  |  |  |  |  |  |  |  |

*Note.* * indicates *p* < .05. ** indicates *p* < .01.

| Table 2.  *Means, Standard Deviations, and Correlations of Control Variables* | | | | | | | | | | | | | | | | | |
| --- | --- | --- | --- | --- | --- | --- | --- | --- | --- | --- | --- | --- | --- | --- | --- | --- | --- |
|  |  |  | M | SD | 1 | 2 | 3 | 4 | 5 | 6 | 7 | 8 | 9 | 10 | 11 | 12 | 13 |
| Improved Target Emotions | | |  |  |  |  |  |  |  |  |  |  |  |  |  |  |  |
|  | 1. | Positive Emotions | 3.08 | .90 |  |  |  |  |  |  |  |  |  |  |  |  |  |
|  | 2. | Negative Emotions | 1.46 | .56 | -.35** |  |  |  |  |  |  |  |  |  |  |  |  |
|  | 3. | Upregulation | 3.83 | .93 | .61** | -.27** |  |  |  |  |  |  |  |  |  |  |  |
|  | 4. | Downregulation | 3.76 | .97 | .50** | -.23* | .61** |  |  |  |  |  |  |  |  |  |  |
| Conversation Intensity | | |  |  |  |  |  |  |  |  |  |  |  |  |  |  |  |
|  | 5. | Meaningfulness (t) | 4.05 | .94 | .40** | -.11 | .30** | .39** |  |  |  |  |  |  |  |  |  |
|  | 6. | Meaningfulness (r) | 4.31 | .80 | -.12 | -.01 | -.08 | -.10 | .18 |  |  |  |  |  |  |  |  |
|  | 7. | Depth (t) | 3.54 | 1.05 | .32** | .03 | .25** | .17 | .46** | .16 |  |  |  |  |  |  |  |
|  | 8. | Depth (r) | 3.77 | 1.05 | -.12 | .07 | -.06 | -.04 | .21* | .58** | .12 |  |  |  |  |  |  |
| Closeness | | |  |  |  |  |  |  |  |  |  |  |  |  |  |  |  |
|  | 9. | Connection (t) | 3.17 | 1.30 | .59** | -.18 | .43** | .38** | .26** | -.11 | .25** | -.01 |  |  |  |  |  |
|  | 10. | Connection (r) | 3.26 | 1.27 | .09 | -.03 | .09 | .11 | .24** | .37** | .19* | .42** | .00 |  |  |  |  |
|  | 11. | Affiliation (t) | 3.50 | .83 | .63** | -.26** | .47** | .45** | .39** | .01 | .28** | -.05 | .59** | .12 |  |  |  |
|  | 12. | Affiliation (r) | 3.57 | .71 | .09 | -.14 | .09 | .06 | .12 | .38** | .09 | .46** | .14 | .50** | .14 |  |  |
| Regulator Empathy | | |  |  |  |  |  |  |  |  |  |  |  |  |  |  |  |
|  | 13. | Empathic concern | 3.87 | 0.63 | .04 | -.00 | .05 | .05 | .11 | .48** | .05 | .41** | -.15 | .33** | .06 | .35** |  |
|  | 14. | Perspective-taking | 3.76 | 0.62 | .08 | -.04 | .03 | -.03 | .01 | .45** | .07 | .30** | -.10 | .31** | .08 | .21* | .55** |

*Note.* Upregulation = regulator success at upregulating target’s positive emotions rated by target, downregulation = regulator success at downregulating target’s negative emotions rated by target, (t) = target-rated, (r) = regulator-rated

Table 3.

*Correlation of Extrinsic Reappraisal with Control Variables*

| Variable | *M* | *SD* | 1 |
| --- | --- | --- | --- |
| Extrinsic Strategy Use |  |  |  |
| 1. Extrinsic reappraisal | 4.67 | 1.01 |  |
| Conversation Intensity |  |  |  |
| 2. Meaningfulness (t) | 4.05 | 0.94 | .15 |
| 3. Meaningfulness (r) | 4.31 | 0.80 | .01 |
| 4. Depth (t) | 3.54 | 1.05 | .15 |
| 5. Depth (r) | 3.77 | 1.05 | .13 |
| Closeness |  |  |  |
| 6. Connection (t) | 3.17 | 1.30 | .09 |
| 7. Connection (r) | 3.26 | 1.27 | .08 |
| 8. Affiliation (t) | 3.50 | 0.83 | .11 |
| 9. Affiliation (r) | 3.57 | 0.71 | .15 |
| Regulator Empathy |  |  |  |
| 10. Empathic concern | 3.87 | 0.63 | .01 |
| 11. Perspective-taking | 3.76 | 0.62 | .09 |
|  |  |  |  |

*Note.* Table 3 contains the correlations between variables other than extrinsic reappraisal.

(t) = target-rated, (r) = regulator-rated

**Examining Alternative Explanations**

Table 4.

*Regressions Accounting for Regulator’s Motivational Bias to Endorse Extrinsic Reappraisal Use*

|  | **Target Negative Emotion** | | | | **Target Positive Emotion** | | | |
| --- | --- | --- | --- | --- | --- | --- | --- | --- |
| Predictors | *β* | SE | *t* | *p* | *β* | SE | *t* | *p* |
| Perceived target downregulation | -.08 | .06 | -1.35 | .18 | .10 | .10 | .82 | .41 |
| Perceived target upregulation | .07 | .06 | 1.15 | .25 | -.06 | .10 | -.62 | .53 |
| Perceived target downregulation X extrinsic reappraisal | .07 | .05 | 1.20 | .24 | .04 | .09 | .42 | .68 |
| Perceived target upregulation X extrinsic reappraisal | -.05 | .07 | -.71 | .50 | .08 | .11 | .72 | .47 |
| **Extrinsic reappraisal** | -.12 | .06 | -2.22 | .03* | .18 | .09 | 2.10 | .04* |
| Fit | *F* | *df* | *p* | R^2^ | *F* | *df* | *p* | R^2^ |
|  | 2.16 | 5,115 | .06 | .05 | 1.92 | 5,115 | .10 | .04 |
| Multicollinearity | VIFs < 1.74 | | | | VIFs < 1.74 | | | |

*Note.* Perceived target downregulation = the extent that the regulator perceived they downregulated the target’s negative emotions, perceived target upregulation = the extent that the regulator perceived they upregulated the target’s positive emotions.

**p* < .05. ** *p* < .01. *** *p* < .001.

Table 5.

*Regressions Accounting for Regulator’s Extrinsic Prohedonic Goal*

|  | **Target Negative Emotion** | | | | **Target Positive Emotion** | | | |
| --- | --- | --- | --- | --- | --- | --- | --- | --- |
| Predictors | *β* | SE | *t* | *p* | *β* | SE | *t* | *p* |
| Extrinsic prohedonic goal | .02 | .06 | .45 | .65 | -.06 | .09 | -.66 | .51 |
| Extrinsic prohedonic goal  X extrinsic reappraisal | -.04 | .05 | -.93 | .36 | .01 | .07 | .21 | .84 |
| **Extrinsic reappraisal** | -.12 | .06 | -2.12 | .04* | .23 | .09 | 2.56 | .01* |
| Fit | *F* | *df* | *p* | R^2^ | *F* | *df* | *p* | R^2^ |
|  | 2.42 | 3,117 | .07 | .03 | 2.55 | 3,117 | .06 | .04 |

*Note.*

**p* < .05. ** *p* < .01. *** *p* < .001.

Table 6.

*Perceived Responsiveness as a Moderator of the Effectiveness of Extrinsic Strategies*

|  | **Target Emotion Improvement** | | | |
| --- | --- | --- | --- | --- |
| Predictors | *β* | SE | *t* | *p* |
| Model I |  |  |  |  |
| Perceived Regulator Responsiveness | .73 | .14 | 5.21 | <.001*** |
| Extrinsic Reappraisal | .36 | .14 | 2.61 | .01** |
| Interaction | -.09 | .13 | -.74 | .46 |
|  |  |  |  |  |
| Fit | *F* | *df* | *p* | R^2^ |
|  | 14.99 | 3,117 | <.001*** | .26 |
| Model II |  |  |  |  |
| Perceived Regulator Responsiveness | 1.17 | .23 | 5.03 | <.001** |
| Extrinsic Acceptance | 1.00 | .71 | 1.41 | .16 |
| Interaction | -.25 | .17 | -1.47 | .15 |
|  |  |  |  |  |
| Fit | *F* | *df* | *p* | R^2^ |
|  | 12.84 | 3,117 | <.001*** | .23 |
| Model III |  |  |  |  |
| Perceived Regulator Responsiveness | 1.27 | .93 | 2.62 | <.001** |
| Extrinsic Acceptance | -.92 | .91 | -1.01 | .31 |
| Interaction | .28 | .22 | 1.27 | .21 |
|  |  |  |  |  |
| Fit | *F* | *df* | *p* | R^2^ |
|  | 13.62 | 3,117 | <.001*** | .24 |
|  |  |  |  |  |

*Note.*

**p* < .05. ** *p* < .01. *** *p* < .001.

Table 7a.

*Regressions Predicting Target Emotions Using all Strategies*

|  | **Target Negative Emotion** | | | | **Target Positive Emotion** | | | |
| --- | --- | --- | --- | --- | --- | --- | --- | --- |
| Predictors | B | SE | *t* | *p* | B | SE | *t* | *p* |
| Extrinsic reappraisal | -.12 | .05 | -2.38 | .02* | .22 | .08 | 2.72 | .008** |
| Extrinsic suppression | -.03 | .06 | -.43 | .67 | -.05 | .10 | -.48 | .63 |
| Extrinsic acceptance | -.07 | .06 | -1.17 | .24 | .09 | .10 | .98 | .28 |
| Gender | -.11 | .10 | -1.11 | .27 | .20 | .16 | 1.26 | .21 |
|  |  |  |  |  |  |  |  |  |
| Fit | *F* | *df* | *p* | R^2^ | *F* | *df* | *p* | R^2^ |
|  | 2.12 | 4,116 | .08 | .04 | 2.67 | 4,116 | .04* | .05 |

Table 7b.

*Regressions Predicting Target-Rated Regulation Success Using all Strategies*

|  | **Downregulating Negative Emotion** | | | | **Upregulating Positive Emotions** | | | |
| --- | --- | --- | --- | --- | --- | --- | --- | --- |
| Predictors | B | SE | *t* | *p* | B | SE | *t* | *p* |
| Extrinsic reappraisal | .20 | .09 | 2.28 | .02* | .32 | .09 | 3.66 | <.001 |
| Extrinsic suppression | .08 | .11 | .70 | .49 | .03 | .11 | .33 | .75 |
| Extrinsic acceptance | .23 | .11 | 2.13 | .04* | .17 | .10 | 1.59 | .11 |
| Gender | .20 | .18 | 1.10 | .28 | .32 | .18 | 1.82 | .07 |
|  |  |  |  |  |  |  |  |  |
| Fit | *F* | *df* | *p* | R^2^ | *F* | *df* | *p* | R^2^ |
|  | 2.73 | 4,116 | .03* | .05 | 4.88 | 4,116 | .001*** | .11 |

Table 8.

*Gender Differences in Extrinsic Emotion Regulation*

|  | Males | Females | Gender difference |
| --- | --- | --- | --- |
|  | Mean (SD) | Mean (SD) | t (*p*) |
| Extrinsic reappraisal | 4.76 (.84) | 4.50 (1.13) | .16 (.40) |
| Extrinsic suppression | 2.82 (.83) | 2.26 (.86) | .55 (<.001***) |
| Extrinsic acceptance | 5.80 (.80) | 6.12 (.69) | .32 (.02*) |
| Goal to upregulate targets’ positive emotions | 4.10 (.53) | 4.10 (.66) | 0 (.94) |
| Goal to downregulate  targets’ negative emotions | 4.19 (.44) | 4.19 (.60) | 0 (.99) |

*Note: All moderation analysis of gender*strategy use on target emotion outcomes were non-significant (p > .11) except for the use of acceptance on targets’ perception of regulator success at reducing their negative emotions (p = .037). In female dyads, extrinsic acceptance was associated with better improvement of target’s negative emotions (as rated by targets).

Table 9a.

*Regressions Predicting Target Emotions After Excluding Participants Without Prohedonic Goals*

|  | **Target Negative Emotion** | | | | **Target Positive Emotion** | | | |
| --- | --- | --- | --- | --- | --- | --- | --- | --- |
| Predictors | B | SE | *t* | *p* | B | SE | *t* | *p* |
| Extrinsic reappraisal | -.14 | .06 | -2.59 | .01* | .26 | .09 | 2.84 | .005** |
| Extrinsic suppression | -.05 | .07 | -.81 | .42 | .05 | .11 | .49 | .63 |
| Extrinsic acceptance | -.06 | .07 | -.85 | .40 | .19 | .11 | 1.76 | .08 |
|  |  |  |  |  |  |  |  |  |
| Fit | *F* | *df* | *p* | R^2^ | *F* | *df* | *p* | R^2^ |
|  | 2.67 | 3,100 | .05 | .05 | 2.67 | 3,100 | .01* | .08 |

Table 9b.

*Regressions Predicting Target Emotions After Excluding Participants Without Prohedonic Goals*

|  | **Downregulating Negative Emotion** | | | | **Upregulating Positive Emotions** | | | |
| --- | --- | --- | --- | --- | --- | --- | --- | --- |
| Predictors | B | SE | *t* | *p* | B | SE | *t* | *p* |
| Extrinsic reappraisal | .20 | .10 | 2.0 | .04* | .32 | .10 | 3.38 | .001** |
| Extrinsic suppression | .17 | .12 | 1.42 | .16 | .14 | .12 | 1.20 | .23 |
| Extrinsic acceptance | .27 | .12 | 2.24 | .03* | .20 | .12 | 1.71 | .09 |
|  |  |  |  |  |  |  |  |  |
| Fit | *F* | *df* | *p* | R^2^ | *F* | *df* | *p* | R^2^ |
|  | 3.27 | 3,100 | .02* | .06 | 5.09 | 3,100 | .003** | .11 |

Table 10

*Regressions Predicting Target Outcomes from Observer-rated Regulator Strategy Use*

|  | **Target Negative Emotion** | | | | **Target Positive Emotion** | | | |
| --- | --- | --- | --- | --- | --- | --- | --- | --- |
| Predictors | B | SE | *t* | *p* | B | SE | *t* | *p* |
| Observer-rated extrinsic reappraisal | -.07 | .04 | -1.92 | .06 | .19 | .06 | 3.25 | .002** |
| Observer-rated extrinsic suppression | .02 | .04 | .52 | .60 | -.04 | .07 | -.50 | .62 |
| Fit | *F* | *df* | *p* | R^2^ | *F* | *df* | *p* | R^2^ |
|  | 2.10 | 2,112 | .13 | .02 | 5.61 | 2,112 | .005* | .07 |

|  | **Downregulating Negative Emotion** | | | | **Upregulating Positive Emotions** | | | |
| --- | --- | --- | --- | --- | --- | --- | --- | --- |
| Predictors | B | SE | *t* | *p* | B | SE | *t* | *p* |
| Observer-rated extrinsic reappraisal | .28 | .06 | 2.89 | .005** | .16 | .06 | 2.70 | .008** |
| Observer-rated extrinsic suppression | .01 | .07 | .11 | .91 | <.001 | .07 | .006 | .99 |
| Fit | *F* | *df* | *p* | R^2^ | *F* | *df* | *p* | R^2^ |
|  | 4.20 | 2,112 | .02* | .06 | 3.67 | 2,112 | .03* | .04 |

|  | **Perceived Regulator Responsiveness** | | | |
| --- | --- | --- | --- | --- |
| Predictors | B | SE | *t* | *p* |
| Observer-rated extrinsic reappraisal | .13 | .04 | 3.16 | .002** |
| Observer-rated extrinsic suppression | -.15 | .05 | -3.06 | .003** |
| Fit | *F* | *df* | *p* | R^2^ |
|  | 10.72 | 2,112 | <.001*** | .15 |
